# The Key Role of Phosphate on Vascular Calcification

**DOI:** 10.3390/toxins11040213

**Published:** 2019-04-09

**Authors:** Mario Cozzolino, Paola Ciceri, Andrea Galassi, Michela Mangano, Stefano Carugo, Irene Capelli, Giuseppe Cianciolo

**Affiliations:** 1 Renal Division, ASST Santi Paolo e Carlo, Department of Health Sciences, University of Milan, 20142 Milan, Italy; andrea.galassi@asst-santipaolocarlo.it (A.G.); michelamangano91@gmail.com (M.M.); 2 Renal Research Laboratory, Department of Nephrology, Dialysis and Renal Transplant, Fondazione IRCCS Ca’ Granda, Ospedale Maggiore Policlinico & Fondazione D’Amico per la Ricerca sulle Malattie Renali, 20122 Milan, Italy; p.ciceri@hotmail.it; 3 Cardiology Unit, ASST Santi Paolo e Carlo, Department of Health Sciences, University of Milan, 20142 Milan, Italy; stefano.carugo@unimi.it; 4 Department of Experimental Diagnostic and Specialty Medicine (DIMES), Nephrology, Dialysis and Renal Transplant Unit, S. Orsola Hospital, University of Bologna, 40138 Bologna, Italy; irene.capelli@gmail.com (I.C.); giuseppe.cianciolo@aosp.bo.it (G.C.)

**Keywords:** hyperphosphatemia, chronic kidney disease, secondary hyperparathyroidism, vascular calcification, phosphate binder

## Abstract

Vascular calcification (VC) is common in dialysis and non-dialysis chronic kidney disease (CKD) patients, even in the early stage of the disease. For this reason, it can be considered a CKD hallmark. VC contributes to cardiovascular disease (CVD) and increased mortality among CKD patients, although it has not been proven. There are more than one type of VC and every form represents a marker of systemic vascular disease and is associated with a higher prevalence of CVD in CKD patients, as shown by several clinical studies. Major risk factors for VC in CKD include: Increasing age, dialysis vintage, hyperphosphatemia (particularly in the setting of intermittent or persistent hypercalcemia), and a positive net calcium and phosphate balance. Excessive oral calcium intake, including calcium-containing phosphate binders, increases the risk for VC. Moreover, it has been demonstrated that there is less VC progression with non-calcium-containing phosphate binders. Unfortunately, until now, a specific therapy to prevent progression or to facilitate regression of VC has been found, beyond careful attention to calcium and phosphate balance.

## 1. Introduction

Cardiovascular disease (CVD) is the most common cause of death in patients with chronic kidney disease (CKD) [1]. The high cardiovascular risk may be due in part to excess vascular calcification (VC), which is observed even in very young dialysis patients, who lack the typical cardiovascular risk factors of hypertension, dyslipidemia, and smoking [2,3,4]. Furthermore, studies suggest that oral calcium intake, particularly by calcium-containing phosphate binders, worsens VC among CKD patients. However, there is no specific therapy to prevent progression, or to facilitate regression of VC, beyond careful attention to calcium and phosphate balance.

There are two types of VC in CKD patients, with different pathogenesis [5,6]: Medial and intimal calcification. Medial calcification occurs as a result of both a phenotype switch of vascular smooth muscle cells (VSMCs) to osteoblast–like cells and local inflammation [5,7,8,9]. The phenotype change is initiated by hyperphosphatemia, hypercalcemia, and, possibly, high concentrations of parathyroid hormone (PTH) [10,11]. Hyperphosphatemia increases activity of the sodium-dependent cotransporters, PiT-1 and PiT-2 [12], which upregulates genes associated with matrix mineralization [5,7,12,13]. Hypercalcemia and hyperphosphatemia both increase the release of matrix vesicles, resulting in the deposition of hydroxyapatite in the extracellular matrix [14,15]. Intimal calcification is secondary to established atherosclerosis. The pathogenesis of atherosclerosis appears to be the same in non-CKD and in CKD patients, even if shear stress, local inflammation, and calcification of VSMC-derived microvesicles, are amplified in CKD patients, so accelerating the calcification process in the intima [16]. Many of the factors that cause medial calcification (hyperphosphatemia, hypercalcemia, and hyperparathyroidism) probably worsen intimal calcification. In addition, the arterial stiffness caused by medial calcification likely directly contributes to shear stress, atherosclerosis, and intima calcification [17].

Fibroblast Growth Factor 23 (FGF23) is a hormone produced by bone cells (both osteoblasts and osteocytes) in response to vitamin D and high phosphate load [18]. Elevated circulating levels of FGF23 lead to phosphate-wasting disorders, and strongly suppresses renal 1α-hydroxylase expression, causing a reduction of the synthesis of the vitamin D hormone 1α,25-dihydroxyvitamin D_3_ in kidney proximal tubules [18]. The FGF23-related suppression of renal 1α-hydroxylase appears to be a physiologically crucial process. Clearly, high circulating calcitriol levels caused by loss of FGF23 function are not detrimental per se, but the toxicity is rather greatly caused by hyperphosphatemia and hypercalcemia, both induced by increased vitamin D activity.

## 2. Definitions and Clinical Significance of VC

Cardiovascular calcification (CVC) associates with several diseases, including end stage renal disease (ESRD) and CVD.

Calcium phosphate deposition, especially in the form of apatite, is the hallmark of CVC and can occur in the blood vessels, myocardium, and cardiac valves. Calcium phosphate deposits are present in distinct layers of the blood vessel and associate with specific pathologies.

So far, no study has systematically evaluated the distribution of CVC at different sites in large CKD populations across stages of disease.

There are four different types of VC [19]: Intimal artery calcification or medial artery calcification, valvular calcifications, and calciphylaxis. Distinguishing medial from intimal calcification is possible with a light microscopic examination: Intimal calcification is disclosed as irregular, discrete, plague-like calcification, whereas medial calcification reveals tram-tract, non-stenotic diffuse calcified wall thickness in radiological and ultrasonographic images. Evaluation of coronary artery calcification (CAC) by computed tomography detects and quantifies vascular and valvular calcifications and is an accepted tool for CVC risk assessment but it is not able to discriminate between intimal and medial calcification.

Medial calcification is an active process detectable in the course of ageing, CKD, and diabetes, and secondary to mineral bone disorders, inflammatory status, humoral factors, and phenotypic switch of resident cells. Compared to calcification of the media layer, current evidence points to a different sequence of events driving calcification in atherosclerotic plaque [20,21,22]. Calcification in the intima appears to be a secondary to inflammation, but still dependent on VSMCs and macrophage infiltration into the early atheromatous lesion and on the effect of humoral factors.

In ESRD patients, both intimal and medial calcification occur, but arterial medial calcification is the most prevalent [5]. Thus, both intimal and medial calcification are probably the major contributors to the 10- to 100-fold increase in cardiovascular mortality risk observed in ESRD patients [17].

**Figure 1:**
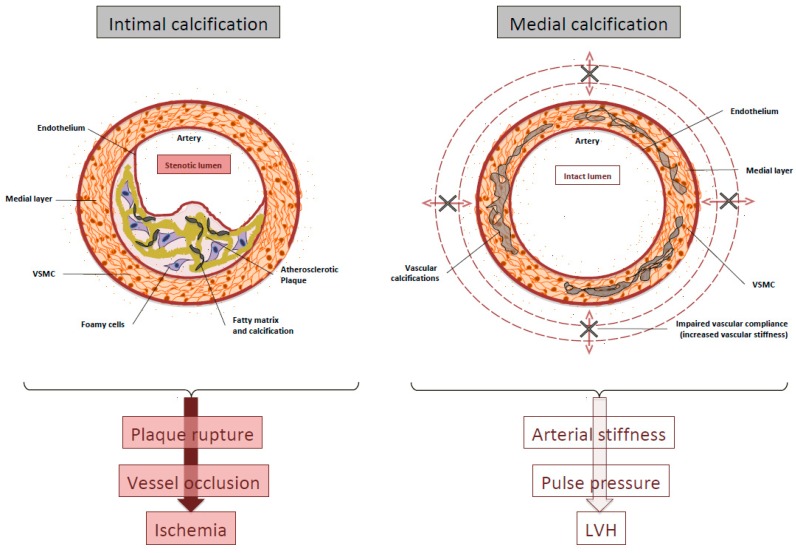
Schematic representation of intimal and medial calcification. LVH: Left ventricular hypertrophy; VSMC: Vascular smooth muscle cells.

The clinical consequences of intimal versus medial layer calcification, although often coexisting in the same patient, may be very different [23,24]. The main consequence of intimal disease is probably plaque rupture and acute vessel occlusion, whereas medial calcification induces arterial stiffness with the consequent increase in systolic blood pressure that contributes to left ventricular hypertrophy (LVH) (Figure 1).

Epidemiological and clinical studies have highlighted an association between serum phosphate levels and VC, even in the normal range at every stage of the renal disease, and also in the general population [25,26,27,28]. Due to these results, and to the findings of experimental models, phosphate is now accepted as a major direct inducer of VC.

To better elucidate the relationship between calcium and phosphate disorders and VC, there are two different mechanisms that are proposed: A passive mechanism, as a direct calcium–phosphate precipitation in the vasculature, and an active process, through the induction of bone-associated genes in VSMCs, that acquire a bone-forming (osteoblast-like) phenotype [6,9].

## 3. VC is An Actively Regulated Process

Growing evidence suggests that CVC is a highly regulated process, involving both inductive and inhibitory processes [29]. Under experimental conditions in vitro, cells from the tunica media (VSMCs, adventitial fibroblasts, and pericytes) switch to osteochondrogenic differentiation and matrix mineralization [30,31]. These studies suggest that cell-mediated processes tightly control procalcific and anticalcific mediators in the artery. The balance between inductors and inhibitors of calcification is fundamental to avoid ectopic calcification and maintain normal vessel physiology, even if the precise role of each inhibitor and inducer still needs deep investigation [32]. Under pathological conditions, this balance is modified, and results in ectopic mineralization.

The hallmark of VC involves both intima and media. It is represented by the activation of cells committed to an osteoblastic program. Cells with an osteogenic phenotype may originate from vascular, wall-resident, mesenchymal stem cells, transdifferentiated mature or circulating VSMCs, pericytes, or circulating calcifying cells (CCCs) [33]. CCCs have been associated with coronary artery disease and calcific aortic stenosis, in both advanced CKD and diabetes [34,35,36]. Although their role is not yet fully defined, the detection and recognition of CCCs is a critical step in the understanding of CVC pathogenesis. However, up to now, there are still limited data available concerning the factors related to the presence of CCCs, and there is no clear proof of CCCs actively participating in vascular medial calcification.

The current major theories to explain VC include: (1) loss of inhibition, (2) induction of osteochondrogenesis, (3) apoptosis, (4) abnormal calcium and phosphate homeostasis, (5) circulating nucleation complexes/paracrine factors derived from bone, and (6) matrix degradation.

Recently, Moe’s research group [37], demonstrated that VSMCs matrix vesicles can be considered as propagator of calcification in non-calcified VSMCs. In fact, VSMCs from aorta of CKD rats are able to secrete microvesicles that induce healthy VSMCs to calcify.

These data confirm a cell–to–cell communication in the pathogenesis of VC and account for the evidence that patients at the beginning of dialysis showing artery calcification, have a more rapid progression of calcification itself [38]. Matrix vesicles were endocytosed by healthy VSMCs, with an increase of intracellular calcium concentration, and specific enzyme pathways were activated.

An important role in the calcification process development is played by the uremic serum milieu with uremic toxins and phosphate as key regulators of VC in ESRD. It has been demonstrated that serum from uremic patients either induces or accelerates calcium deposition in vitro due to the presence of a complex miscellaneous of uremic factors [39,40,41]. Recently, it has been shown that there is a significant positive correlation between the degree of VC in vivo and the serum calcific potential, in high-Pi condition, in vitro through a higher potential to induce VSMCs osteoblastic trans-differentiation, apoptosis, and necrosis [42].

## 4. VSMCs: A Model System to Investigate Mechanisms of VC In Vitro

VSMCs classically exhibit a contractile phenotype and reside in the media of blood vessels. These cells highly express genes that are required for the maintenance of myofilament function and structure. VSMCs phenotype is characterized by the ability to reversibly enter in a synthetic state of production and proliferation of large amounts of extracellular matrix. Therefore, VSMCs can be activated from a quiescent, differentiated state into an actively synthesizing and proliferating phenotype. This phenotypic change associates with reduction of smooth muscle cell markers and can be induced by several stimuli in vitro, including mechanical stress, injury, and growth factors [3]. This finding, however, is controversial as synthetic VSMCs may derive from vascular stem cells (VSCs) found in all three layers of the vascular wall. After vascular injury, medial mature VSMCs die while VSCs differentiate into synthetic VSMCs or other cell types contributing to vascular repair or lesion progression [43] Whatever the cell population from which synthetic VSMCs derive, chronic, low-grade inflammation, oxidative stress, and mineral bone disorders —hallmarks of CKD—are also strong inducers of VSMCs dysfunction.

Once a synthetic phenotype is aquired, the VSMCs switch their morphology, proliferate, start to migrate, and synthesize enormous amounts of extracellular matrix components leading to arterial remodelling. For the repair of vascular damage, these functional changes are essential [44], but VSMCs may also induce the development of several vascular diseases, including hypertension, atherosclerosis, and CVC. Recently, the role of endothelium has been found to significantly contribute to VC through endothelial-mesenchymal transitions [45] In particular, endothelial cells (ECs) line the vessel walls and are in contact with blood, displaying heterogeneity in their response to exogenous stimuli. These ECs play an important role in the homeostasis of the vascular system, and when ECs are exposed to various environments, undergo dynamic phenotypic switching, which might result in EC dysfunction, causing several diseases. Recent studies show the importance of endothelial to mesenchymal transition in endothelial dysfunction during inflammation, leading to pathological states, such as renal fibrosis, pulmonary arterial hypertension, VC, and atherosclerosis. Interestingly, cinacalcet, a calcimimetic agent, reduces synthesis and secretion of PTH and decreases serum levels of phosphate, suppresses the switch of ECs into mesenchymal cells, leading to attenuation of cardiac fibrosis [46].

## 5. The Role of Phosphorus in the Development and Progression of VC

In the United States there are about 25 million subjects affected by CKD stages 2 to 5, and between them, the risk of CVD is linked to a very high mortality risk. Furthermore, patients with higher risk of mortality are those with advanced CKD. In fact, ‘chronological’ age in a 70-year-old patient with ESRD corresponds to a ‘biological’ age of a 90-year-old subject with normal renal function [3]. Clearly, the traditional risk factors for CVD cannot fully explain the higher increased cardiovascular risk in CKD.

In the CKD population, CVC is associated with several non-classical risk factors that may be unique to CKD [47]. One such factor is high serum phosphorus levels, which has been linked to CVC in several preclinical and clinical studies and is emerging as a central regulator of extra-skeletal calcification in the CKD population [7,8,9].

**Figure 2:**
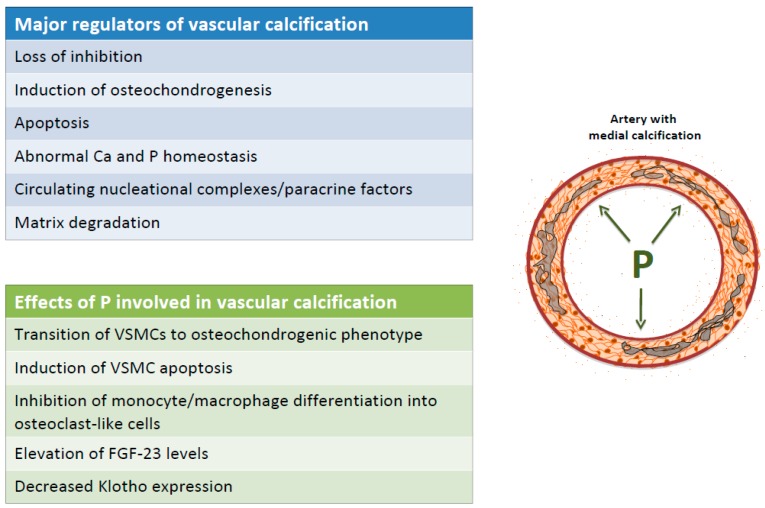
Major regulators and effects of P on vascular calcification. Ca: calcium; FGF-23: fibroblast growth factor-23; P: phosphate; VSMC: vascular smooth muscle cells.

VC is an early feature of CKD patients and its progression is linked to high phosphate load and hyperphosphatemia. It appears to be involved in several mechanisms that promotes and triggers VC progression, including: (1) Transition of VSMCs from a contractile to an osteochondrogenic phenotype and VSMCs extracellular matrix mineralization; (2) induction of VSMCs apoptosis; (3) inhibition of monocyte/macrophage differentiation into osteoclast-like cells; (4) increased fibroblast growth factor 23 (FGF23) levels; (5) reduction in Klotho expression (Figure 2).

Several in vitro studies have been performed using VSMCs to determine whether phosphorus directly affects VC. The well-known association between hyperphosphatemia and VC in CKD patients, can be elucidated in the experimental setting, in which VSMCs are exposed to high levels of inorganic phosphate, considering the calcification process induced in the extracellular matrix surrounding VSMCs [48,49]. The master gene for bone differentiation, named Cbfa-1 or RUNX2 (core binding factor A1), drives the entrance of phosphate into the cytosol of VSMCs, inducing bone matrix protein production in the extracellular matrix.

Similar to bone calcification, extra-skeletal calcification can be obtained by molecular and cellular mechanisms, including matrix vesicles and bio-apatite synthesis [50]. Furthermore, phosphate directly induces phenotypic switches in VSMCs, causing them to transform from a contractile phenotype into an osteochondrogenic phenotype [51]. In vitro studies have examined how phosphate can induce a change in phenotype of VSMCs; for example, cultured human aortic smooth muscle cells switch to osteoblastic cell types at different levels of extracellular phosphate, showing a dose dependent increase in calcium deposition into the extracellular matrix. Similarly, in a study of bovine aortic VSMCs, mineralization in culture was associated with the tremendous loss of smooth muscle-specific gene expression in the presence of an organic phosphate donor, β-glycerol phosphate [52]. Interestingly, protection from high-phosphate induced smooth muscle marker down-regulation in VSMCs reduces calcification [53].

Thus, during CKD, phosphate load increases and accumulates outside bones, determining the fact that phosphate can be considered a ‘uremic toxin’. Of course, the effects of phosphate on biological systems are extremely important, but the adverse effects of hyperphosphatemia in CKD should be emphasized, even in early stages of the disease. Therefore, in combination with several factors, hyperphosphatemia can cause damage in several cells and tissues, such as bones, parathyroid glands, and, most importantly, the heart and vessels. In fact, hyperphosphatemia strongly associates with vascular and valvular calcification, arteriosclerosis, and an increased risk of cardiovascular death, especially in advanced CKD patients.

## 6. Phosphate-Induced Apoptosis of VSMCs

Some studies support a link between VSMCs apoptosis and VC, suggesting that apoptosis is a key regulator of VSMCs calcification [32,54,55]. Proudfoot et al. [56] reported that, in a human in vitro VSMCs calcification model, apoptosis occurred before calcium deposition and demonstrated that inhibiting apoptosis inhibits calcification, whereas stimulating apoptosis increases calcification. In 2008, Shroff et al. [57] studied the effects of dialysis treatment on accelerated atherosclerosis and arteriosclerosis, in part by triggering smooth muscle cell apoptosis. Interestingly, VSMCs in vessels of dialysis patients showed increased apoptosis and dramatic damage. In this study, they hypothesize that calcium accumulation in the vessel begins in response to increased levels of calcium and phosphate. Moreover, they hypothesize that protective mechanisms, such as the maintenance of adequate mineralization inhibitor levels and extrusion of intracellular calcium via vesicle release, preserves normal VSMCs function.

In the dialysis milieu, uremic toxins and the continued exposure to high-Pi lead to apoptosis. This, in turn, increases local Ca^++^ deposition and reduces levels of VSMC-derived mineralization inhibitors, which potentiates osteo/chondocytic differentiation of VSMCs. Apoptotic cells form a nidus for calcification, releasing apoptotic bodies loaded with calcium that start hydroxyapatite deposition in the extracellular matrix and thus the calcification process [58].

However, Shanahan et al. [11] found that the molecular and cellular mechanisms of VC in the experimental model of VSMCs does not require apoptosis for the initiation of calcification. Nonetheless, once calcium phosphate crystals are deposited in the matrix, the calcification process is accelerated by increased apoptosis. It has been demonstrated that VSMCs are able to be phagocytosed by both human and synthetic atherosclerosis-derived calcium phosphate crystals, resulting in a rapid increase in intracellular calcium concentrations and consequent inflammation and apoptosis [59].

Recently, Shanahan et al. [11] hypothesized a close interplay between apoptotic bodies formation in VSMCS and phosphate-induced cell phenotype switch. Contractile VSMCs adapt to the hostile conditions by undergoing lineage switch to a synthetic bone forming phenotype. These cells are able to secrete matrix vesicles, in the attempt to avoid calcium overload. On the contrary, VSMCs that fail to differentiate go to apoptosis, which also results in budding of matrix mineralization and vesicles. Both pathways lead to extracellular calcium phosphate deposition, via vesicle release with enhanced risk of apoptosis of surrounding VSMCs [11].

## 7. Phosphate and Autophagy in VSMCs

Autophagy, a highly regulated and dynamic process of self-digestion, seems to play a central role in VSMCs mineralization. The term ‘autophagic cell death’ has been widely used to indicate a type of cell death that is accompanied by massive vacuolization of the cytoplasm [3]. However, the relationship between cell death and autophagy remains controversial. This evidence suggests that, although cell death can occur together with autophagy, the latter likely represents a pro-survival mechanism activated by dying cells, trying to cope with stress [8,9].

Dai et al. [60] demonstrated that, although the inhibition of autophagy reduced phosphate-induced VSMCs apoptosis, it caused an increment in calcium deposition in both animal (bovine and rat VSMCs) and human aortic cells. Moreover, their results showed that the induction of autophagy is correlated with a decrease of calcification in bovine VSMCs and rat aortic rings. Thus, autophagy may be an endogenous protective mechanism counteracting phosphate-induced VC not acting by the decrease of VSMCs apoptosis, but acting by the reduction of matrix vesicle release.

## 8. Iron, Phosphate, and VC

One of the main debated issues regarding VC is the possibility to block and eventually reverse already established VC in order to reduce CVD morbidity and mortality in CKD patients.

Two new aluminum–calcium-free iron-based phosphate binders are now available to treat hyperphosphatemia in CKD, namely sucroferric oxyhydroxide and iron citrate [61]. On the other hand, iron deficiency anemia represents a serious cardiovascular complication in the CKD population. Iron deficiency is under-diagnosed and, consequently, under-treated in CKD. The idea to have iron-based phosphate binders beyond anemia treatment may help to counteract CVD burden in CKD. First, the pathogenesis of iron deficiency anemia has been explored in this population, and it is recognized that impaired intestinal absorption of dietary iron, chronic inflammation, reduced dietary iron intake, blood loss, and increased iron requirements during therapy with erythropoietin, play a crucial role. Clinical guidelines for anemia include: The use of iron in CKD population, with several suggestions and only a few recommendations [62].

Recently some in vitro data on the effect of iron on VSMCs calcification has been published [63,64]. These in vitro studies attempt to clarify the direct effect of iron on the pathological pathways of VC, independently of the iron phosphate binding effect. The same effect of calcium deposition prevention by iron in a model of high-phosphate challenge has been demonstrated, both on human and rat VSMCs. The two groups investigated different aspects of the VC process. It has been found that the increased expression and ferroxidase activity of the ferritin heavy chain prevents VC by an inhibition of VSMCs osteoblastic transformation [65]. Furthermore, the calcification inhibition by iron is also driven by the prevention of apoptosis and the enhancement of autophagy. These data account for iron having an impact in preventing high-Pi induced calcium deposition in vitro. However, interestingly, iron, added when the calcification is already there, in a protocol called ‘therapeutic’ similar to ‘clinical practice’, has the ability to completely stop the high-phosphate induced time-dependent calcium deposition [64]. This result is highly surprising, considering that after 7 or more days of high-Pi challenge the VSMCs that receive iron are already transformed in simil-osteoblasts and actively deposit calcium-phosphate crystals. However, in the ‘prophylactic’ approach, when VSMCs cells receive high-phosphate and iron, their phenotype is muscular, and iron may act on the prevention of high-phosphate induced VC mechanisms. Thus, with ‘therapeutic’ administration of iron in vitro, it is possible to completely block calcium deposition, even if VSMCs are already transformed in simil-osteoblasts and actively deposit calcium hydroxyapatite.

Considering that the main detrimental effect of VC is the altered stiffness of the vessel wall, another important aspect in vascular calcification is probably played by the extracellular matrix (ECM) characteristics modification.

A recent study by Ciceri et al. [64], showed that iron citrate is able to redirect ECM characteristics towards muscular ECM if administered when calcification is already established. In fact, a ’therapeutic’ addition of iron, besides blocking the osteo-chondrogenic shift of granules and inducing no additional deposition of acidic mucins typical of cartilage and bone, was able to protect the aortic wall from the progression of high-Pi induced fibrosis development, promoting an apparent fibril rearrangement and block of high-Pi induced thickening of collagen fibrils. Interestingly, iron was also able to induce an improvement in the elastic structure of the vessel wall through the protection of the aortic wall from high-Pi induced progression of elastinolysis and to revert it. In order to understand the effect of intravenous administration of iron in CKD patients at different stages, new research will clarify the pathways underlying the role of iron in an in vivo model of VC. Interestingly recent evidence shows that it may be possible to block calcium deposition and partially revert some VC aspects, at least in vitro.

## 9. Phosphate and VC in the Healthy Population

Two prospective studies of large cohorts of healthy adults have demonstrated that phosphate levels at the upper limit of the normal range, and in any case >3.9 mg/dL, are independently associated with a greater likelihood and a more rapid progression of, coronary artery calcium.

In the first study, an association between serum phosphate levels and coronary artery calcification was evaluated in 3015 individuals enrolled in the Coronary Artery Risk Development in Young Adults (CARDIA).

The presence of CAC, assessed by computed tomography 15 years later, was found to be significantly associated with serum phosphate levels [25].

In the second study, each 1-mg/dl increase in phosphorus imparted odds ratios for CAC of 1.61 (incidence) and 1.54 (prevalence), risks comparable to traditional CVD risk factors. Unfortunately, no indexes of mineral metabolism, above all, PTH, vitamin D levels, and FGF23, were evaluated in neither study [66].

In 1938 individuals enrolled in the cardiovascular health study, Linefsky and his group observed an association between serum phosphate levels and cardiac valve calcification in a population-based cohort with normal renal function. Importantly, associations were not observed between PTH, calcium, or 25-OHD levels and calcification outcomes [26].

## 10. Phosphate and VC in CKD

Adeney et al. studied more than 400 multi-ethnic participants with mild to moderate CKD, with no clinical evidence of CVD. These authors reported that each 1 mg/dL (0.32 mmol/L) increase in phosphate level is independently and strongly associated with an higher prevalence of coronary artery, thoracic, aortic valve, and mitral VC [27].

In a CRIC (Chronic Renal Insufficiency Cohort) study, more than 1000 CKD patients were investigated for presence of VC. In fact, CAC progression was associated with the following disorders: Mineral metabolism, inflammation indexes, and kidney function. The annual change in CAC score was significant for p values above 3,9 mg/dL. Moreover, higher CAC score significantly associated with both high serum PTH and FGF23 levels [67].

These findings were not confirmed in a further analysis of more than 500 CKD patients without clinical signs of CVD enrolled in the MESA (Multi-Ethnic Study of Atherosclerosis) study, in which CAC was measured at baseline and after 19 and 38 months. In this study, the presence of diabetes has been described as a significant predictor of incidence of CAC, while the male gender has been identified as a significant predictor of progression of CAC [68].

Many researchers are looking for serum biomarkers to identify VC patients. Very recently a new test, named maturation time (T50) of calciprotein particles in serum, has been proposed as a novel in vitro blood test that should provide a propensity score to extra-skeletal calcification. This T50 test is based on considering the difference in timing of the change of calcium phosphate–containing primary CPP to hydroxyapatite–containing secondary CPP. Both forms of CPP are formed in vitro on the addition of very high concentrations of buffered calcium and phosphate solutions to serum of patients [69]. The balance of either initiating or inhibiting factors into each serum sample rules the transformation time (serum calcium phosphate precipitation time [T50]).

High serum calcification propensity (i.e., reduced serum T50), defining the overall tendency to calcify, associates with progressive aortic stiffening, and is, therefore, a predictor of poor survival.

In a prospective cohort of almost 200 patients with early CKD stages (3 and 4), with a median of 5.3 years of follow-up, the authors described how baseline T50 independently associated with aortic pulse wave velocity in all randomized patients, while in about 50% of CKD patients, progressive aortic stiffening was associated to T50 after 30 months of follow-up. Indeed, the lowest T50 was principally dependent on serum phosphate levels and serum Fetuin-A levels [70].

## 11. Phosphate and VC in ESRD

VC is significantly associated with the cardiovascular prognosis of ESRD patients [71,72]. In a study conducted on 237 patients on hemodialysis and peritoneal dialysis, hypercalcemia and secondary hyperparathyroidism, unlike hyperphosphatemia, associated with an increased risk of progression of aortic calcification [73]. Another study on aortic calcification, included 184 peritoneal dialysis /haemodialysis patients and found that dialysis vintage, the baseline calcification score, and the presence of diabetes associated with progression of CVC, without derangement of biomarkers of mineral metabolism [74].

On the other hand, Shang et al., in 207 adult patients on peritoneal dialysis, found that high serum phosphate levels and aging are independent risk factors for CAC progression and that high body mass index shows a trend of increased prevalence in peritoneal dialysis patients. Moreover, serum phosphate levels are positively associated with nutritional biomarkers and peritoneal dialysis adequacy, indicating that a high daily phosphate intake and peritoneal dialysis inadequacy may accelerate CAC progression [28].

FGF23 is a major regulator of phosphorus metabolism in health and disease, and its synthesis is stimulated by the expansion of the organic phosphate pool. Serum FGF23 levels increase very early during CKD, considering the late development of hyperphosphatemia. Thus, in CKD, both high FGF23 and PTH levels are considered very sensitive and the earliest markers of deregulated phosphate homoeostasis.

It has been described by Nasrallah et al. that serum FGF23 was independently associated with calcification of the aorta in patients on dialysis not affected by diabetes. These authors studied how serum phosphate levels did not correlate with CVC, but highlighted an interesting interaction between serum FGF23 levels and aortic calcification index, independent of serum phosphate levels [75]. In agreement with this report, Ozkok et al., in 74 haemodialysis patients, found that the association between serum FGF23 levels and progression of CAC score was independent of serum phosphorus levels [76]. In logistic regression analysis, the higher coronary artery calcification progression was significantly associated with serum FGF23, phosphorus levels, and baseline coronary artery calcification score.
